# Factors Associated with Participation in Pulmonary Tuberculosis Screening Using Chest X-Ray among Diabetes Mellitus Type II Patients in Denpasar, Bali, Indonesia

**DOI:** 10.1155/2018/9285195

**Published:** 2018-03-20

**Authors:** I Gusti Ngurah Edi Putra, Putu Ayu Swandewi Astuti, I Ketut Suarjana, Ketut Hari Mulyawan, I Made Kerta Duana, Ni Made Dian Kurniasari, I Wayan Gede Artawan Eka Putra

**Affiliations:** School of Public Health, Faculty of Medicine, Udayana University, Denpasar, Indonesia

## Abstract

Diabetes mellitus (DM) increases the risk of developing pulmonary tuberculosis (TB) disease. Therefore, pulmonary TB screening among DM patients is essential. This study aimed to identify factors associated with participation of DM type II patients in pulmonary TB screening using chest X-ray. This was a cross-sectional analytic study and was part of TB-DM screening study in Denpasar, Bali, Indonesia. The sample consisted of 365 DM type II patients selected by quota sampling among DM type II patients joining the screening program from January until March 2016 in 11 public health centres in Denpasar. Data were collected via structured interviews. The contributing factors were determined by modified Poisson regression test for cross-sectional data. From the findings, less than half (45.48%) of DM type II patients participated in chest X-ray examination for TB. Factors associated with participation in pulmonary TB screening were having a higher educational level [APR = 1.34, 95% CI (1.07–1.67)], having family member who developed pulmonary TB disease [APR = 1.47, 95% CI (1.12–1.93)], the travel time to referral hospital for screening being ≤ 15 minutes [APR = 1.6, 95% CI (1.26–2.03)], having health insurance [APR = 2.69, 95% CI (1.10–6.56)], and receiving good support from health provider [APR = 1.35, 95% CI (1.06–1.70)]. Therefore, training for health provider on providing counselling, involvement of family members in screening process, and improving the health insurance coverage and referral system are worth considering.

## 1. Introduction

Tuberculosis (TB) is widely recognised as a leading cause of death [1–3]. The Global Tuberculosis Report 2017 published by World Health Organization (WHO) estimated that 10.4 million people developed active TB disease in 2016. Around 1.3 million deaths due to TB were estimated among HIV-negative people whilst 374,000 TB deaths occurred among people living with HIV. The percentage of new cases varied by WHO region where Southeast Asia and Africa were the most contributed regions, accounting for 45% and 25%, respectively [3].

Diabetes mellitus (DM) was identified as a risk factor of TB [4]; studies showed that DM increases the likelihood of developing TB disease and vice versa [5, 6]. Globally, in 22 countries with the highest burden of TB, the prevalence of DM ranged from 2 to 9% among the general population [7]. According to WHO, 8 of 10 countries with the highest incidence of DM were also stated as high burden of TB [8]. The study conducted by Lin et al. about pulmonary TB screening among DM patients in China showed that case notification rate (CNR) of pulmonary TB among DM patients was higher, ranging from 334 to 804/100,000, compared to the general population at 78/100,000 people [9].

Indonesia as a developing country is the second highest country in the world on the number of TB incident as high as 1.02 million people, contributing to almost 10% of global new TB cases in 2016 [3]. Ministry of Health (MoH), Indonesia, estimated that the prevalence rate of TB in the last two years, 2015 and 2016, was 643 and 628/100,000 people, respectively [10]. Meanwhile, the prevalence of DM was also high according to the national survey,* Riset Kesehatan Dasar *(National Health Research) conducted by MoH; the prevalence among 15+ years old increased from 5.7% in 2007 to 6.9% in 2013 [11]. International Diabetes Federation (IDF) currently positions Indonesia as the sixth country of DM cases, estimation of 10.3 million people [12].

Unfortunately, the prevalence of pulmonary TB disease among DM patients has not been well-documented and reported in Indonesia. It was suggested that the escalation of pulmonary TB cases among DM patients also occurred in line with the increasing prevalence of DM in Indonesia [13]. The recent study conducted by Livia et al. showed 28% of 738 DM patients screened in Hasan Sadikin Bandung Hospital were active TB cases and 9.3% had history of previous TB disease [14]. Therefore, performing pulmonary TB screening among DM patients is important to increase CNR of TB and to provide adequate treatment for both TB and DM.

Detecting pulmonary TB disease among DM patients has not been routinely conducted. Hence, it will require intention and active participation from DM patients to access pulmonary TB screening services. There is no study to date in Indonesia exploring factors associated with the participation of DM patients in pulmonary TB screening. This participation in pulmonary TB screening falls into health-seeking behaviour practices which can be influenced by individual factor and more complex factors such as the interaction to the environmental factors [15].

MoH, Indonesia, has developed an algorithm of pulmonary TB screening among DM type II patients [16]. This algorithm was pilot tested in Denpasar, Bali. The study was started in January 2016 in eleven public health centres (PHCs) in Denpasar. This study was initiated by researchers from School of Public Health, Faculty of Medicine, Udayana University, in collaboration with Regional Health Office of Denpasar. Based on this algorithm, the screening was conducted among DM type II patients. Chest X-ray examination was proposed as screening method since collecting sputum was not produced by almost all DM type II patients. This study was part of this screening pilot study which aimed to identify factors associated with participation in pulmonary TB screening using chest X-ray among DM type II patients.

## 2. Methods

### 2.1. Study Setting and Participants

The study was conducted in Denpasar city administration (capital of Bali, Indonesia). This city has 4 subcities with 11 PHCs. This was a cross-sectional analytic study and under the umbrella of TB-DM screening study conducted in 11 PHCs from January to March 2016. The inclusion criteria were DM type II patients undergoing the treatment in PHCs in Denpasar, aged 15 years and over, and receiving counselling for pulmonary TB screening using chest X-ray delivered by the health providers. Those who were under TB treatment were excluded from this study.

### 2.2. Sample Size Determination

The estimation formula for two population proportions was employed with an assumption of 95% confidence interval, 80% power, 54.6% as the estimation of people who participate in pulmonary TB screening (p2) based on the study by Sañé Schepisi et al. about tuberculosis case finding among immigrants, refugees, and asylum seekers [17], 69.6% (15% higher than p2) as the estimation of DM type II patients who have good knowledge will participate in pulmonary TB screening (p1), and 10% nonresponse rate. Thus, the total sample size was 365.

### 2.3. Sampling Technique, Data Collection Instrument, and Procedures

The study involved all of 11 PHCs in Denpasar where DM type II patients were undergoing their treatment and 2 public hospitals which are on the referral system for performing pulmonary TB screening using chest X-ray. The PHCs in Denpasar are not equipped to provide chest X-ray services; thus referral is needed. The research teams had more than one meeting and workshop with the head of all PHCs and representatives from hospital to introduce the screening algorithm including the referral systems of DM type II patients from PHCs to the selected hospital. Therefore, it prevents the possibility of DM type II patients who undergo chest X-ray at unrecommended hospitals.

The sample was collected proportionally using quota sampling technique based on the number of DM type II patients participating in pulmonary TB screening program from January to March 2016 in 11 PHCs. DM type II patients who came to PHCs were counselled by assigned health provider about the screening and were facilitated to make the decision regarding pulmonary TB screening. Those who were willing to participate in the screening would be referred to the selected hospital. The patients must show the screening result from the hospital to the health provider at the PHCs in the following days in order to be defined as participating in the screening. Meanwhile, DM type II patients who either decided to not participate in the screening after the counselling or did not participate in the screening after they had been referred to the hospital were included for not participating in pulmonary TB screening.

Trained enumerators were stationed in each piece of PHCs collected DM type II patients' data via interviews using structured questionnaires after the counselling had been delivered by health providers. The questionnaires were tested to 5% of intended sample size among DM type II patients who had similar characteristics with the targeted sample in two PHCs in Badung district, located near Denpasar city. The data collected for this study were including the participation in pulmonary TB screening using chest X-ray examination as the dependent variable whereas the independent variables consisted of sociodemographic characteristics (age, sex, educational level, employment status, and per capita income), pulmonary TB history (their own history of suffering pulmonary TB in the past and the family member who developed pulmonary TB diseases), access to the referral hospital for screening (in terms of distance and travel time to referral hospital and type of health care financing), patients' knowledge and attitude, and support from the health provider. In addition, after the interview process had been finished, the enumerators remained to stay regularly in the PHCs to monitor the progress of pulmonary TB screening among the respondents.

### 2.4. Data Analysis and Presentation

Descriptive statistics such as percentage were used to describe variables. The contributing factors to the participation in pulmonary TB screening were determined by modified Poisson regression test for cross-sectional data. Results were presented with proportion (%), prevalence ratio (PR), 95% confidence interval (CI), and *p* value. In the multiple Poisson regression models, the final model was considered at *p* value ≤ 0.05.

### 2.5. Ethical Consideration

This study has been approved by Ethics Committee of Development and Research Unit, Faculty of Medicine, Udayana University, and Sanglah General Hospital. Written informed consent forms were obtained from all participants involved in this study before face-to-face interview.

## 3. Results

Table 1 shows that, from 365 DM type II patients as the respondents, most of them aged more than 45 years (91.78%), half patients were female (52.05%) and completed low educational level (52.33%), and majority were employed (65.48%). The per capita income was grouped into two categories based on poverty line defined by Statistics Centre Board for the urban area in Bali province where poverty line in 2015 was 341,554 rupiahs [18], resulting in most respondents earned per capita income above the poverty line (78.27%). There were only 20 (5.48%) who had a history of previous pulmonary TB disease and 42 (11.51%) had family member who developed pulmonary TB disease.

Regarding the geographical access, more than half (56.44%) of respondents resided within 5 kilometres or less from the referral hospital and 51.78% required travel time less than or equal to 15 minutes from home to the referral hospitals. The majority of respondents (92.60%) were covered by health insurance scheme, which includes the coverage for chest X-ray for pulmonary TB screening. Unfortunately, most of them had poor knowledge toward TB-DM comorbidity (83.84%) and negative attitude toward pulmonary TB screening (61.10%). In addition, most of them have received good support from health provider (55.62%).

Table 2 shows only 166 (45.48%) of the respondents participated in pulmonary TB screening. Of the remaining 199 (54.52%) who did not participate in the screening, 106 (29.04%) refused to participate soon after the counselling and 93 (25.48%) cancelled to participate after being referred. The various reasons for not participating in pulmonary TB screening were busy schedule; thus they did not have time for the screening (61.31%), the cost of screening was expensive (4.02%), none of the family members could accompany them to the referral hospital for screening (18.59%), and travel distance to the referral hospital was so far (3.52%). Moreover, there were 16.08% respondents who refused to participate because they did not have TB symptoms and some others (3.52%) were afraid of the undesirable screening outcome.

The multivariate analysis using modified Poisson regression test for cross-sectional data at Table 3 showed that the factors associated with participation in pulmonary TB screening among DM type II patients were completing higher educational level [APR = 1.34, 95% CI (1.07–1.67)], having family member who developed pulmonary TB disease [APR = 1.47, 95% CI (1.12–1.93)], the travel time to referral hospital for screening being ≤ 15 minutes [APR = 1.6, 95% CI (1.26–2.03)], having health insurance [APR = 2.69, 95% CI (1.10–6.56)], and receiving good support from health provider [APR = 1.35, 95% CI (1.06–1.70)].

## 4. Discussion

Sociodemographic factors such as educational level and having family member who developed pulmonary TB disease were associated with the uptake of pulmonary TB screening. DM type II patients in this study who completed high education level were more likely to participate in the chest X-ray examination. Higher education level plays an important role in the ability to receive new information and to make an informed decision. Educational level as a significant determinant of health-seeking behaviour has been well established. A study conducted by Fagundez et al. reported that educational level was a proximate determinant of the adherence of TB treatment [19]. This finding is also supported by a theory proposed by Green and Kreuter which confirmed that participation in pulmonary TB screening as one of health-seeking behaviour practices can be determined by the predisposing factors such as sociodemographic characteristics and knowledge or attitude [15].

Another theory of health behaviour such as Health Belief Model highlights that the individual behaviour is driven by the beliefs or perceptions [20]. DM type II patients who had a family member history of developing pulmonary TB disease were more likely to be exposed to TB information. It will eventually shape their perception of susceptibility to being infected with TB and the seriousness of the disease because of their household contact history. People who experienced a close connection to pulmonary TB are more likely to have higher concern of their risk and to accept the information around TB screening provided by the PHCs staff during the counselling [21]. Thus, exploring the history of TB disease among family member and other closed contacts during counselling process is important to leverage the participation in the screening.

Geographical access such as distance to referral hospital was a significant barrier to participate in pulmonary TB screening. The previous study conducted by Adenager et al. about factors associated with treatment delay among pulmonary TB patients showed that TB sufferers who experienced the distance more than 2.5 km to TB treatment facilities would be delayed to have treatment 1.6 times compared to those who travelled in less than 2.5 km [22]. Another study also found the similar finding where the defaulting TB treatment was associated with the distance and the time taken to get the hospital [23]. The far distance remains a barrier to reach health care facilities since it requires the high cost of transportation [24, 25].

The financial issue was also hampered pulmonary TB screening uptake among DM type II patients. It revealed that the ownership of health insurance increased the likelihood of the participation in pulmonary TB screening. This is similar to the study published by Bourne which found that the engagement in health insurance scheme affected the health status and health care utilization patterns [26]. In this study, the cost of chest X-ray examination in referral hospital was covered by the health insurance; hence this screening should be accessible for people with low income as long as they bore the government's health insurance. Therefore, a small proportion of DM type II patients (4.02%) who did not participate in the screening and argued the expensive cost of screening might have not been included in health insurance scheme.

The last factor contributing to the participation in pulmonary TB screening in this study was the support from the health provider. Health providers play a significant role in providing adequate health information [27]. They might contribute to improve the knowledge, attitude, and decision-making process of DM type II patients. In this study, although most DM type II patients received good support in terms of information availability through the counselling process, the knowledge and attitude were poor. This might be linked to the counselling process provided by health providers which probably is more directive approach to participating in the screening rather than providing enough information to develop the knowledge and attitude. It creates the situation where DM type II patients performed the expected behaviour, but they still had poor knowledge and negative attitude.

Those findings suggest the need to improve the knowledge and attitude of DM type II patients through improvement on the counselling quality. The health providers must be trained prior to the counselling to make them capable of providing the information effectively and facilitating the decision-making process properly. In addition, the engagement of family members during the counselling process is important since they can give support and address the potential barriers. Sufficient social support for patients to overcome the barriers may influence their decision [28]. Family members can provide the transportation support especially if the distance to referral hospital is relatively far and financial support to whom did not have health insurance. Furthermore, expanding the health insurance coverage should also be considered to increase the screening uptake. In addition, improving the referral system by involving all hospitals in providing chest X-ray services is worth considering to increase the screening uptake.

## 5. Conclusions

The proportion of DM type II patients in Denpasar who participated in pulmonary TB screening was less than half (45.48%). The factors associated with participation in pulmonary TB screening among DM type II patients were having a higher educational level, having a family member who developed pulmonary TB disease, travel time to referral hospital being less than or equal to 15 minutes, having health insurance, and receiving good support from health providers. Providing the counselling training is essential for health providers to build their capability in delivering the information and facilitating the decision-making process. In addition, family members should be involved to address potential barriers by providing the transportation and financial support. Increasing health insurance coverage and referral system of the screening are substantial to enhance the participation of DM type II patients in pulmonary TB screening.

## Figures and Tables

**Table 1:** Sociodemographic characteristics, access, knowledge, attitude, and support from health provider, Denpasar, January–March 2016.

Variable	*n* = 365	%
Age		
≤45 years old	30	8.22
>45 years old	335	91.78
Sex		
Male	175	47.95
Female	190	52.05
Educational level		
Higher educational level	174	47.67
Lower educational level	191	52.33
Employment status		
Employed	239	65.48
Unemployed	126	34.52
Per capita income		
≤poverty line	68	21.73
>poverty line	245	78.27
History of suffering TB		
Yes	20	5.48
No	345	94.52
Family history of suffering TB		
Yes	42	11.51
No	323	88.49
Distance to referral hospital		
≤5 kms	206	56.44
>5 kms	159	43.56
Travel time to referral hospital		
≤15 minutes	189	51.78
>15 minutes	176	48.22
Type of health care financing		
Health insurance	338	92.60
Out of pocket	27	7.40
Knowledge		
Good	59	16.16
Poor	306	83.84
Attitude		
Positive	142	38.90
Negative	223	61.10
Support from health provider		
Good	203	55.62
Poor	162	44.38

TB: tuberculosis; kms: kilometres.

**Table 2:** The overview of DM patient's participation in pulmonary TB screening Denpasar, January–March 2016.

Variable	*n* = 365	%
Participation in pulmonary TB screening		
Yes	166	45.48
No	199	54.52
(i) Refused after the counselling	106	29.04
(ii) Did not participate in the screening after	93	25.48
being referred		
Reasons for not participate (*n* = 199)		
(i) Afraid if the result is positive	7	3.52
(ii) Do not have time for screening	122	61.31
(iii) Expensive cost	8	4.02
(iv) There is no family member who can	37	18.59
accompany to referral hospital		
(v) Far distance to referral hospital	7	3.52
(vi) Do not feel the TB symptoms	32	16.08
(vii) No reason	8	4.02

TB: tuberculosis.

**Table 3:** Analysis of factors influencing participation in pulmonary TB screening among DM type II patients, Denpasar, January–March 2016.

Variable	Bivariable analysis			Multivariable analysis		
	CPR	95% CI PR	*p* value	APR	95% CI PR	*p* value
Age						
≤45 years old	1.03	0.59–1.78	0.920			
>45 years old	Ref					
Sex						
Male	1.03	0.76–1.40	0.826			
Female	Ref					
Educational level						
Higher educational level	1.47	1.08–1.99	0.014	1.34	1.07–1.67	0.009
Lower educational level	Ref			Ref		
Employment status						
Employed	1.19	0.85–1.65	0.304			
Unemployed	Ref					
Per capita income						
≤poverty line	1.14	0.75–1.72	0.534			
>poverty line	Ref					
History of suffering TB						
Yes	1.11	0.58–2.09	0.758			
No	Ref					
Family history of suffering TB						
Yes	1.36	0.89–2.09	0.153	1.47	1.12–1.93	0.006
No	Ref			Ref		
Distance to referral hospital						
≤5 kms	1.65	1.88–2.28	0.003			
>5 kms	Ref					
Travel time to referral hospital						
≤15 minutes	1.73	1.26–2.39	0.001	1.6	1.26–2,03	<0.001
>15 minutes	Ref			Ref		
Type of health care financing						
Health insurance	3.24	1.19–8.72	0.02	2.69	1.10–6.56	0.029
Out of pocket	Ref			Ref		
Knowledge						
Good	1.39	0.95–2.01	0.086			
Poor	Ref					
Attitude						
Positive	1.15	0.84–1.56	0.389			
Negative	Ref					
Support from health provider						
Good	1.49	1.08–2.04	0.015	1.35	1.06–1.70	0.013
Poor	Ref			Ref		

TB: tuberculosis, kms: kilometres, CPR: crude prevalence ratio, APR: adjusted prevalence ratio, and CI: confidence interval.
